# Neural Network-Based Routing Energy-Saving Algorithm for Wireless Sensor Networks

**DOI:** 10.1155/2022/3342031

**Published:** 2022-07-01

**Authors:** Lili Pang, Jiaye Xie, Qiqing Xu

**Affiliations:** Industrial Center, Nanjing Institute of Technology, Nanjing 211167, China

## Abstract

With the evolvement, standards have changed, mobile Internet technology has also been upgraded, and it has also driven the development of smart objects mobile. With the continuous development of smart objects mobile, the bottleneck of small node size and low battery energy storage has not been solved in the end, which makes the research of wireless sensor network energy-saving technology become the focus, and the improvement of routing technology is an effective way to improve energy-saving technology. From the data transmission energy consumption of smart objects mobile, the routing algorithm of smart objects mobile is discussed and analyzed and the classical representative LEACH is the object of in-depth research. Routing algorithms can easily and reliably process network data and make the network work well and are widely used in highly secure military systems and smaller commercial networks. Aiming at these deficiencies, a corresponding improved algorithm is proposed, and it is tested through simulation and specific experiments to verify the correctness and the system's reliability. The SMPSO-BP algorithm converges when the number of iterations is about 600, which is earlier than the LEACH algorithm and the improved LEACH algorithm, so the SMPSO-BP algorithm is due to the other two algorithms. In the wireless sensor network routing energy consumption experiment, in addition, the SMPSO-BP algorithm uses less energy than the other two methods. Therefore, the energy-saving algorithm under the neural network data fusion mechanism is still feasible.

## 1. Introduction

The smart objects mobile is the main path of data transmission in the Internet of Things, along with the advancement of Internet, which is the main support for data transmission, and it is also the function that consumes the most energy in the wireless sensor network, which directly affects the overall performance of the entire network. As a new generation of communication technology integrating embedded computing, sensor, network, and wireless communication, wireless sensor network has been widely used in various fields. With information science's rapid growth, wireless sensors with the ability to obtain information from multiple technologies have been applied in various fields of intelligent data monitoring and are favored by various fields because of their low cost and multifunctionality.

The wireless sensor routing energy-saving algorithm aims at delaying the network service life under the premise that the node energy is exhausted and can undertake the network protection function. With the vigorous development of information technology, data transmission has become an indispensable part. The network routing is the carrier of data transmission, in order to improve the efficiency of data transmission and reduce energy consumption during the transmission process. According to the LEACH algorithm, the shortcomings of energy saving in wireless sensors are analyzed, and the redundancy of the original data collection by the sensor nodes is solved to boost downlink costs and decrease wireless carbon emissions using CNNs data space technology.

The combination of neural network and wireless sensor network is used to realize the data fusion in the network. First, the LEACH algorithm is a classic hierarchical routing algorithm used to stabilize the data transmission of wireless sensors, and then an improved LEACH algorithm is introduced on the basis of stable data transmission, and the improved LEACH algorithm is used to find the best path in the data transmission process. Finally, using the powerful platform complex utilization only fits the standards of cooperative sensing processes that are performed, improves the data transmission intelligence level of wireless sensors, reduces energy consumption, and prolongs service life.

## 2. Related Work

Wireless sensors have become one of the research hotspots because of their flexibility and high-precision monitoring capabilities. Dey et al. designed a home version of a wireless ECG monitoring system using Zigbee technology to monitor people's health at home, and the real-time monitoring system records, measures, and monitors ECG activity while maintaining consumer comfort [1]. Jamhour et al. proposed a new SDWSN control plane based on the Constrained Application Protocol to provide comprehensive specifications for the control plane, including the communication infrastructure, control plane protocols, and basic network functions in the controller [2]. Aarti and Anuj discovered the optimum path to transfer packet data from node to receiver, which includes communications systems, core router interfaces, and basic network activities in the computer. Aarti and Anuj considered the wide range of applications and delicate environments inside which WSNs are used, wherein transmission is as crucial as fuel economy, whereas the best route focused simulated annealing, simulated annealing, and efficient heuristic XGBoost have just been offered to increase delivery ratio [3]. These studies can analyze the relevant conditions of wireless sensors, but the research on sensor life and energy consumption needs to be improved.

With the increase of the number of distributed sensor nodes, researches on energy saving and consumption reduction of wireless sensors appear one after another. Song et al. used the Wasser procedure to execute data mining technique and conversation amongst sink nodes, guaranteeing that each node operates as a member node with quite an additional option, allowing the cable network nodes to spend energy more equitably [4]. Bhola et al. proposed LEACH, a modular technology that changes IoT devices with sensor nodes (CH), which analyzes and organizes data before sending it to the destination node to identify the best path [5]. In order to provide interesting solutions to problems related to agricultural resource optimization, decision support, and land monitoring, Khriji et al. proposed a localization-based RSSI algorithm to determine node locations and developed a low energy adaptive clustering approach adaptive neurofuzzy inference to minimize energy usage across all sink node [6]. In order to realize the efficient and optimal transmission of renewable energy power generation in the energy router, Guo et al. proposed a minimum loss routing algorithm based on the matchmaking tradeoff competition mechanism for the optimal selection of the existing ER transmission path [7]. Liu et al. proposed a Chaos Elite Eco-Evolutionary Algorithm (CENEA) for low-power clustering in EMWSNs for environmental monitoring. Results have shown that CENEA balances node energy and improves node energy usage efficiency, improves accuracy, and minimizes computation time [8]. The research on these energy-saving algorithms is instructive to a certain extent, but in some cases, the demonstration is not sufficient or accurate enough and can be further improved.

## 3. Energy-Saving Algorithm for Wireless Sensor Network Routing

### 3.1. LEACH Algorithm

**Figure 1:**
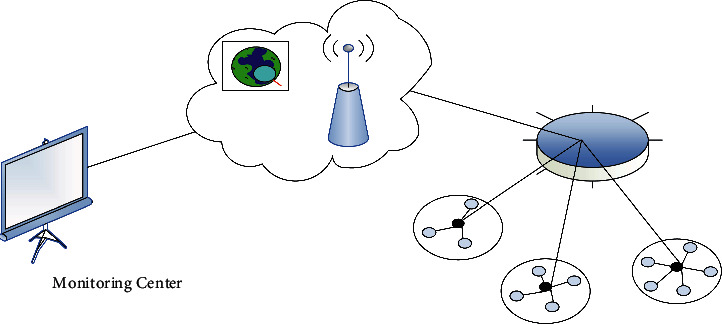
Hierarchical routing.

The Scheme (LEACH) is a minimal flexible grouping routing mechanism that was the first to be published in sensor nodes [9]. The protocol is executed cyclically in units of rounds. Other ordinary nodes select the cluster head closest to themselves and join it to complete the establishment of the cluster; entering the stable data communication stage, the ordinary node transmits the collected data to the cluster head of the cluster where it belongs. After receiving the data, the cluster head completes the fusion operation and finally sends it directly to the sink node [10]. After a round of execution, waiting for a period of time, the network enters the next round of work cycle. The LEACH cluster routing algorithm mainly has two stages: the cluster establishment stage and the stable operation stage. The group building cycle primarily focuses on cluster setup, while the good performance of the proposed level mostly focuses on transmission, as shown in Figure 1. As one of the most classical routing algorithms, LEACH algorithm has some shortcomings, but its proposed hierarchical structure and round-robin mechanism have great reference value.

The LEACH algorithm is a hierarchical routing algorithm, and it is also the most classic one in hierarchical routing. Hierarchical routing algorithms have become a hot research direction of routing algorithms due to their high scalability, high energy utilization, and easy data fusion. The LEACH algorithm solves the stress problem by evenly dispersing the entire platform's electricity cost with each including to [11]. Hierarchical routing is shown in Figure 1. The algorithm randomly selects the cluster head and splits the cluster in a random cycle. This method of selecting cluster heads counterbalances the consumption of energy of nodes and improves the longevity of the network to some extent [12]. Energy saving, network lifetime, data fusion, and scalability are compared across three different topologies, as shown in Table 1.

**Figure 2:**
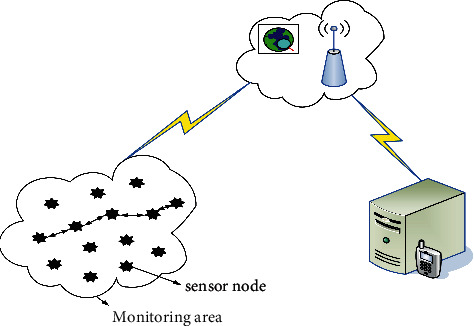
Wireless sensor network architecture.

As shown in Table 1, the Layered Routing algorithm outperforms the other two on all counts [13]. The sensor nodes perform real-time monitoring, information acquisition, and information processing functions on the monitoring object. The collected data is transmitted back to rendezvous junction, which processes the digital information and sends it to the user through the network, thus achieving the purpose of environmental monitoring, target tracking, and data collection [14]. Sensor nodes, sink nodes, and task management nodes form a wireless sensor network system. The traditional wireless sensor network structure is shown in Figure 2.

The routing computing capability of the node also needs to depend on the computing unit. The wireless communication module is composed of the network, MAC, and transceiver and mainly realizes the data communication function: the power supply module supplies energy for the entire sensor node [15].

**Figure 3:**
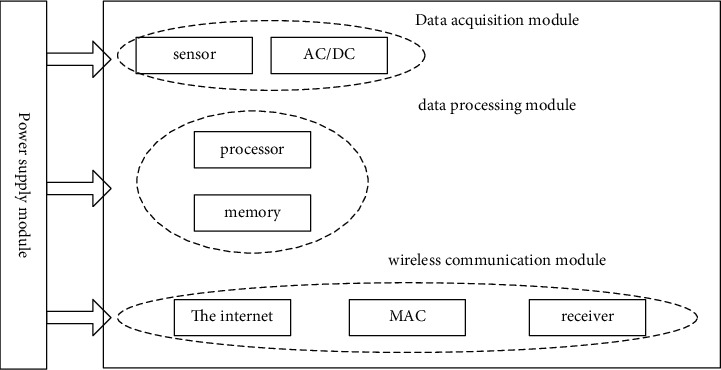
Sensor node block diagram.

As can be seen from Figure 3, the power module is the most important module, and all other modules need the support of the power module. However, most power modules use batteries for energy supply, and the energy is limited, so an effective routing mechanism needs to be designed to lessen the power expenditure of the web and extend the lifespan of the web [5].

**Figure 4:**
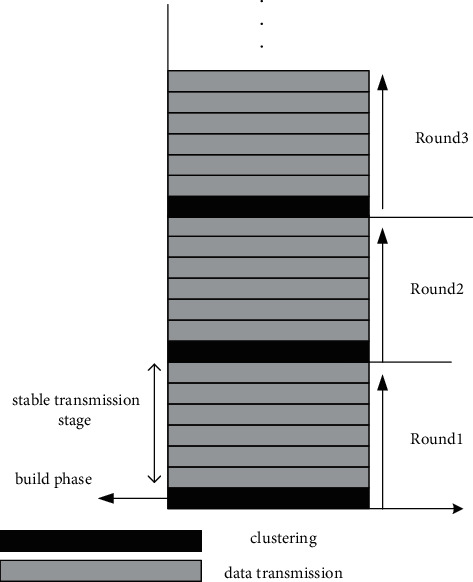
LEACH algorithm run cycle diagram.

The advantages of trunking over flat roaming solutions in saving energy are reflected in the following points: the cluster member nodes simply send the data to the club leaders of the clusters after sensing such traffic. The upper layer backbone network consisting of club leader completes the long distance transmission of data to the sink nodes. The communications modalities can be turned off by the member nodes for the majority of the transmission time [16]. This working mode not only ensures the data communication in the entire network, but also greatly reduces the communication volume in the web and less power hungry. The nodes that constitute the wireless sensor network are usually deployed randomly, and closer to the point where the data gathered by the knots may be relatively resemblance. Meanwhile, the cluster topology is well expandable and applies to massive linear wireless transceiver systems. No matter what type of routing protocol is used, the purpose is to minimize the effort of energy overhead for data transmission and to extend the longevity of the system [17]. The running “round” of the LEACH algorithm is shown in Figure 4.

In the initialization phase of wireless sensor network data transmission, each node generates a random value of [0,1], and the node compares the random value generated by itself with the threshold. If the randomly selected value is less in value than the quota, which will be picked for container leaders. The formula is as follows:

(1) $$\begin{array}{c} T\left(i\right)=\left\{\begin{array}{c} \frac{z}{1-z\times \left(r\times \mathrm{mod}\left(1/z\right)\right)},\;i\in G,\\ 0,\;\text{else}. \end{array}\right. \end{array}$$

In WSN, the functions of sensor nodes are mainly to perceive, process, and transmit data. Since the power expenditure for performing data feeds is quite different from that of other functions, the energy consumption analysis of WSN only considers the communication power generation. The communication energy consumption of wireless sensor network increases with the increase of distance, so the data transmission route has a great influence on the energy consumption of the sensor. Correlation between communication distance *Y* meters and the energy consumption *X* bit generated by the generated data is expressed as the following formula:

(2) $$\begin{array}{c} Q_{ch}\left(X,Y\right)=\left\{\begin{array}{c} XQ_{\text{elec}}+X\varepsilon_{fs}Y^{2},\;Y\leq D_{\text{threshold}},\\ XQ_{\text{elec}}+X\varepsilon_{ch}Y^{4},\;Y>D_{\text{threshold}}, \end{array}\right. \end{array}$$

(3) $$\begin{array}{c} y_{\text{threshold}}=\sqrt{\frac{\varepsilon_{fs}}{\varepsilon_{ch}}}, \end{array}$$

(4) $$\begin{array}{c} Q_{RX}\left(X\right)=XQ_{\text{elec}}, \end{array}$$

(5) $$\begin{array}{c} Q_{AG}\left(X\right)=XQ_{Ya}. \end{array}$$

Here, *Q*_*Ya*_ represents the circuit loss energy of fusing unit-bit data [18]. Therefore, the transmission route has a great influence on the power generation speed of the sensor web, and it is very important for the wireless sensor network to design an efficient route to reduce the power expenditure of the web [19].

### 3.2. Improved LEACH Algorithm

The improved LEACH algorithm introduces the concept of temporary tuft leader head and final tuft leader in the first round of tuft head election. All temporary tuft leader located within the same cluster radius competes for the ultimate tuft leader, avoiding the phenomenon of multiple tuft leader within a tuft radius, and uniformizing the distribution of tuft leader [20]. Compared with the LEACH algorithm, the improved algorithm calculates the optimal number of cluster heads based on the energy characteristics of the base station, so that the cluster heads are distributed relatively uniformly in the node area. The energy threshold is judged during the rotation between the cluster heads within the cluster, and the energy consumption is effectively reduced through multihop transmission between the cluster heads to the base station.

Assuming that each node cluster is a circle with the tuft leader as the center, according to the network model of the algorithm, if the area of the node distribution area is m^2^, the sum of knots is *N*, and the proportion of cluster heads is *p*, then there are *N*_*p*_ nodes in the network. If the clusters are to cover the entire node distribution area, the coverage radius of each cluster needs to be satisfied. *R* is also the shortest distance between cluster head nodes [21].

(6) $$\begin{array}{c} T=\frac{P}{\sqrt{Qn\pi }}, \end{array}$$

(7) $$\begin{array}{c} Q_{ab}=Qn. \end{array}$$

On average, there are (*Q*/*Q*_*ab*_) nodes in each cluster in the network, including *Q*/*Q*_*ab*_ − 1 cluster head and *q* member nodes [22]. The receiving node receives data, fuses data, and sends data. Let *N*_*to*  sin  *k*_ be the distance from the tuft leader to the flume knot, and then power expenditure of the tuft leader is

(8) $$\begin{array}{c} W_{ab}=k\left(\frac{Q}{Q_{ab}}-1\right)W_{elec}+k\frac{Q}{Q_{ab}}W_{DA}+kW_{elec}+k\varepsilon_{mp}d_{to\;\sin\;k}^{4}. \end{array}$$

During a single cluster in each round, the member knots in a single climate only need to transmit water to the leader of the climate. If *Y*_totoch_ is the distance from the member node to the leader of the climate, the power expenditure of the member node is

(9) $$\begin{array}{c} W_{cm}=kW_{elec}+k\varepsilon_{fs}Y_{\text{totoch}}^{2}. \end{array}$$

It is assumed that the coverage area of each cluster is a swarm leader focused on a swarm with a root size of approximately of $R/\sqrt{Q_{ab}\pi }$. If the distribution density of nodes that are part of a fleet of node is *∂*(*x*, *y*), the expectation of the square of the distance from any member node in the circular cluster to the center of the circle is

(10) $$\begin{array}{c} W\left[Y_{\text{toch}}^{2}\right]=\iint \left(x^{2}+y^{2}\right)\partial \left(x+y\right)YxYy, \end{array}$$

(11) $$\begin{array}{c} W\left[Y_{\text{toch}}^{2}\right]=\iint r^{2}\partial \left(r,\theta \right)YrY\theta , \end{array}$$

(12) $$\begin{array}{c} W\left[Y_{\text{toch}}^{2}\right]=\partial \int_{0}^{2\pi }\int_{0}^{\left(R/\sqrt{Q_{ch}\pi }\right)}r^{3}YrY\theta , \end{array}$$

(13) $$\begin{array}{c} W\left[Y_{\text{toch}}^{2}\right]=\partial \frac{R^{4}}{2Q_{ch}^{2}\pi }. \end{array}$$

If the nodes are evenly distributed throughout the network area, then

(14) $$\begin{array}{c} \partial =\frac{1}{\left(R^{2}/Q_{ch}\right)}, \end{array}$$

(15) $$\begin{array}{c} W\left[Y_{\text{toch}}^{2}\right]=\frac{R^{2}}{2Q_{ch}\pi }, \end{array}$$

(16) $$\begin{array}{c} W_{cm}=kW_{\text{elec}}+k\varepsilon_{fs}\frac{R^{2}}{2\pi Q_{ch}}. \end{array}$$

Then, the power expenditure of a single cluster in each round is

(17) $$\begin{array}{c} W_{\text{cluster}}=W_{ch}+\left(\frac{Q}{Q_{ch}}-1\right)W_{ch}\approx W_{ch}+\frac{Q}{Q_{ch}}kW_{cm}. \end{array}$$

Then, the energy consumption of the whole network in each round is

(18) $$\begin{array}{c} W_{wsn}=Q_{ch}W_{\text{cluster}}=Q_{ch}\left(W_{ch}+\frac{Q}{Q_{ch}}W_{cm}\right), \end{array}$$

(19) $$\begin{array}{c} W_{wsn}=k\left(2QW_{\text{elec}}+QW_{DA}+Q_{ch}\varepsilon_{mp}Y_{to\;\sin\;k}^{4}+\frac{\varepsilon_{fs}}{2\pi }\frac{QR^{2}}{Q_{ch}}\right). \end{array}$$

In order to find out what value *Q*_*ch*_ takes, *W*_*wsn*_ is the smallest; let *W*_*wsn*_ take the first derivative of *Q*_*ch*_, and let (*YW*_*wsn*_/*YQ*_*ch*_)=0, and it can be obtained that

(20) $$\begin{array}{c} \varepsilon_{mp}Y_{to\;\sin\;k}^{4}=\frac{\varepsilon_{fs}}{2\pi }\frac{QR^{2}}{Q_{ch}}. \end{array}$$

Then, the best amount of tuft leaders in terms of the number of tuft tips can be solved for

(21) $$\begin{array}{c} Q_{ch}=\frac{Q}{Y_{to\;\sin\;k}^{2}}\sqrt{\frac{Q\varepsilon_{fs}}{2\pi \varepsilon_{mp}}}. \end{array}$$

It can be seen from the formula that the optimal number of tuft tips *Q*_*ch*_ is only related to parameters *ε*_*fs*_ and *ε*_*mp*_, the size of the node distribution area *R*, the total number of nodes *Q*, and the distance *Y*_*to*  sin  *k*_ from the tuft tip to the sink node. The cluster heads generated by the LEACH random method cannot guarantee their uniform distribution. The information collected by these cluster heads has large redundancy, and the transmission of redundant data is a waste of the limited energy of nodes. Therefore, in the network initialization stage, the ideal quantity of pack leader in the web can be figured out according to the following formula parameters, and an appropriate number of cluster heads can be selected in the first round of cluster head election. In the cluster routing mechanism, the data collected by the member nodes are collected and merged by the cluster head before being forwarded, which reduces the data redundancy and reduces the traffic.

### 3.3. SMPSO-BP Data Fusion Algorithm

A neural web (NN), also known as an armed artificial network (ANN), is a process that simulates the thinking of the human brain through extensive interconnections. With the powerful error resilience and the ability of adaptive self-learning, self-organization, and self-adaptation, neural networks model sophisticated affine line mappings and can meet the processing demands of data fusion technology, which is especially suitable for sensor networks. Wireless sensor networks need to solve the problems of limited network energy supply, data transmission quality and network data transmission security in the process of mass data transmission, the ability of utilizing the system's signal manipulation function and auto-reasoning capabilities of neural net. Data fusion is realized, and the development of wireless sensor network application field is also promoted, so the emergence of artificial neural network data fusion technology is inevitable.

The basic idea of data fusion is to correlate and analyze the sensing data from multiple sensor nodes, filter invalid information, and extract feature information and combine it into a data packet of the same size with high accuracy. The reduction of data traffic means the reduction of the amount of data sent or received by sensor nodes, which can effectively extend the network life cycle. The total of packets are received at the sink node satisfactorily. Sink node is used as the basis for measuring the data traffic in the network. The comparison of this data can not only reflect the role of data fusion in wireless sensor network routing, but also reflect the difference in data volume control.

Neural network has the ability of parallel processing, distributed storage, and self-learning for a large amount of data, especially suitable for the stage where data needs to be processed at the same time and can solve the problem of inaccurate information processing. The main idea of the SMPSO algorithm is to mutate some particles whose fitness is too low in the early stage. The way is to update the position of the particle to be the average value of the position of the particle with the highest fitness value, thereby reducing the diversity of the population. Then, the particle calculation formula in the SMPSO algorithm is used to update each particle, and the optimal particle obtained after training is the optimal parameter output. Among them, the fitness value function *f*(*x*) is the mean square error generated in the sample training, as shown in the following formula:

(22) $$\begin{array}{c} k\left(x\right)=\frac{1}{1+\left(1/2n\right)\sum_{b=1}^{n}\left(y_{b}-t_{b}\right)}. \end{array}$$

The standard deviation of the output sequence of the hidden layer node *i* is calculated according to the formula given below, and the correlation between the *i*th neuron node and the jth neuron node in the hidden layer is calculated.

(23) $$\begin{array}{c} f_{t}^{2}={\sum_{b=1}^{n}\left(V_{ib}-{\bar{V}}_{i}\right)}^{2}, \end{array}$$

(24) $$\begin{array}{c} C_{ij}=\frac{\sum_{b=1}^{n}\left(V_{ib}V_{jb}-n{\bar{V}}_{j}{\bar{V}}_{i}\right)}{f_{i}f_{j}}. \end{array}$$

Here, *V*_*ib*_ is the outcome value of the ith node of the hidden layer for sample *p*, and ${\bar{V}}_{i}$ is the average value of *V*_*ib*_. If *C*_*ij*_ > *h*_1_, *f*_*i*_ > *h*_2_, *f*_*j*_ > *h*_2_; delete neuron *j* and modify the connection weight *w*_*ei*_ and threshold *θ*_*e*_ of any neuron *e* in the next layer, as shown in the following formula:

(25) $$\begin{array}{c} w_{ei}=w_{ei}+aw_{ei}, \end{array}$$

(26) $$\begin{array}{c} \theta_{e}=\theta_{e}+b\theta_{e}. \end{array}$$

The wireless sensor network has limited energy, and the communication energy consumption is the main energy consumption of the network. The routing algorithm determines the stability of the entire network. The data fusion model based on BP neural web includes a BP web optimization algorithm based on optimized PSO, namely, the design of the SMPSO-BP algorithm, and it is used to implement the data merge course of the nodes in the wireless sensor web cluster.

## 4. Routing Test of Wireless Sensor Network Based on Neural Network

### 4.1. Test Parameter Settings

Before testing the relevant functions of the wireless sensor on the relevant algorithm, the preparation work should be done first, and the relevant parameters should be set:

1. The sensor node does not move, and it will enter the state of death when its energy is exhausted.
2. In the entire wireless sensor network, there is only one sink node, that is, the sink node, and this node has unlimited energy and computing power and does not need to consider energy supply and node death.
3. Sensor nodes have unique IDs and can communicate with each other.
4. The energy depletion model for text wireless transmission uses the WSN channel energy depletion model, and the node distance is set as the experimental approximate value of 90 m. The sensor knots are strategically located in the measurement area and have the same transmission range. The specific parameters are shown in Table 2.

### 4.2. Node Test Comparison of Related Algorithms

**Figure 5:**
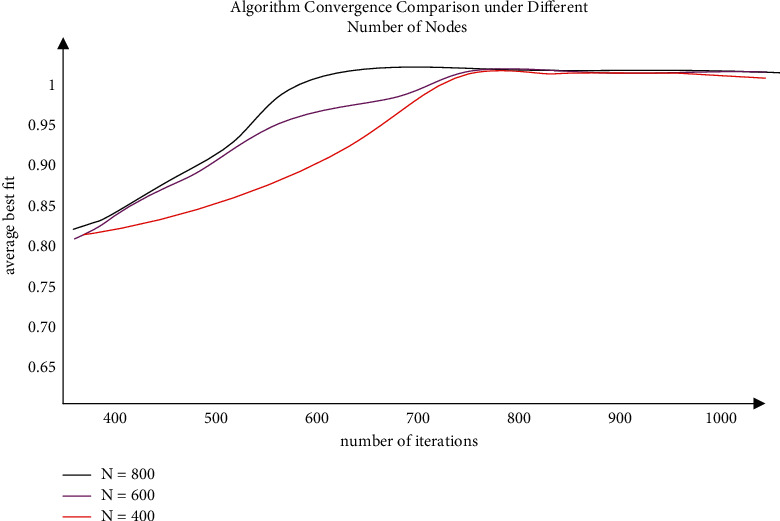
Convergence speed of different algorithms at the same number of nodes and different number of nodes.

The convergence rate, which can be expressed by the average best fitness and the average number of iterations, is used to demonstrate the effect of the SMPSO-BP algorithm's search performance. The average number of iterations will be 100, based on previous experience and experimental testing conditions.  Figure 5 shows the SMPSO-BP calculations for verification with the LEACH calculations and the improved LEACH calculations under the experimental parameter settings. We get the verification of the SMPSO-BP algorithm's convergence simulation results when the number of sensor nodes is 600, 700, and 800, respectively. The curve of the SMPSO-BP algorithm first reaches the level, as shown in the graph above, when the number of knots is 800, indicating that the individual particle swarm obtains the best fitness value first. The scenario with 700 points comes next, followed by the scenario with 600 points. As a result, the SMPSO-BP algorithm proposed in the paper can be applied not only to small-scale nets, but also to massive systems, with a relatively fast aggregation speed.

It is seen from Figure 5 that, by increasing the frequency of repeated generations, the fitness value is constantly approaching the optimal fitness value, and finally the average fitness value is close to 1, which is stable, and the curve converges. This shows that it is practicable to integrate and combine neural neck networks with SMPSO-BP algo for data fusion in radio signal systems. Further, the SMPSO-BP algorithm can arrive at the sweet fitness value much earlier compared to the improved LEACH method; that is, the optimal reading value of the BP neural network can be obtained faster. It can also be seen from the figure that the curve of the original LEACH algorithm without optimization is the slowest, which shows that the LEACH algorithm does have the problem of slow convergence. The other two algorithms with intelligent optimization have a significantly higher curve convergence rate than the LEACH algorithm. And the improved LEACH algorithm converges when the number of evolution iterations is about 700, while the SMPSO-BP algorithm converges, and when the total number of iterations is about 600, this suggests that the speed of convergence of the SMPSO-BP algorithm is indeed faster than that of the improved LEACH algorithm.

The SMPSO-BP algorithm obtained from the experimental analysis is due to the other two algorithms, but at the expense of simulation time. Through 60 independent simulation experiments, the average operation time of SMPSO-BP algorithm, LEACH algorithm, and improved LEACH algorithm is obtained. The results are shown in Table 3.

Compared with the GABP algorithm, the BP network optimization algorithm requires longer operation time, and the BP algorithm without any optimization measures requires the shortest operation time. Although the SMPOS-BP algorithm achieves the optimization of the BP network at the expense of extending the operation time, from the results in the above table, the extended time is relatively within an acceptable range. Therefore, in general, for the application of data fusion in wireless sensor networks, the SMPSO-BP algorithm has shown good performance in terms of efficiency and accuracy.

**Figure 6:**
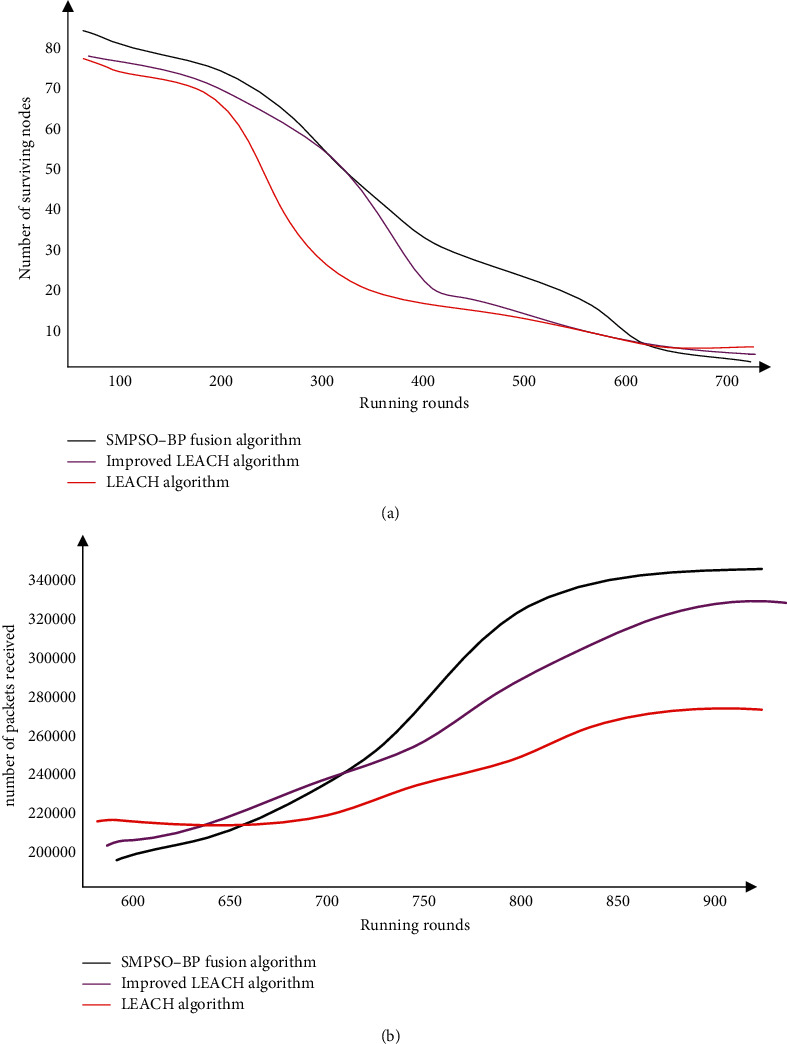
Survival number of network nodes with different algorithms. (a) Comparison of the number of surviving nodes in the network. (b) Comparison of the amount of data received by the node.

The survival rate of sensor nodes is crucial to the normal operation of wireless sensor networks. Once a node enters a state of death due to energy exhaustion or other failures, the node cannot perform the task of sensing and collecting data. Therefore, after the network has been running for a period of time, the comparison and analysis of the number of surviving nodes are an important part of examining the performance of the improved data fusion algorithm. The SMPSO-BP algorithm, the LEACH algorithm, and the improved LEACH algorithm are simulated and compared, and the results are shown in Figure 6.

The curve of the LEACH protocol is greater than the previous version in the early stages of network operation than in the other two cases, as shown in Figure 6. This is due to the fact that the data fusion mechanism is not constrained, so it is sent directly to the socket head point node. The node survival rate drops dramatically as the number of data transmissions grows too large. When the data fusion mechanism is implemented in a network where the SMPSO-BP operator performs the SMPSO-BP approach to processing, the processed data is sent to the repository junction nodes in a stable manner as the amount of transmitted data grows. Because the amount of redundant data is reduced, and the nodes' energy consumption is low, environmental monitoring and data transmission can still be carried out normally, especially at a later stage. The fact that the number of packets received by the water sink nodes increased even after the death of a large number of sensor nodes shows that data fusion can reduce data traffic in the network effectively. During this time, the optimized LEACH protocol also played a supporting role, helping achieve the goal of extending the network's working time.

### 4.3. Comparison of Energy Consumption in the Network

In order to understand and solve the problem of energy consumption of routing nodes in wireless sensor networks, this paper uses SMPSO-BP algorithm, LEACH algorithm, and improved LEACH algorithm to calculate the change of the remaining energy of the whole network in the base station node area and outside the area with time, to understand the energy consumption of the wireless sensor network routing nodes by the neural network of the data fusion technology.

**Figure 7:**
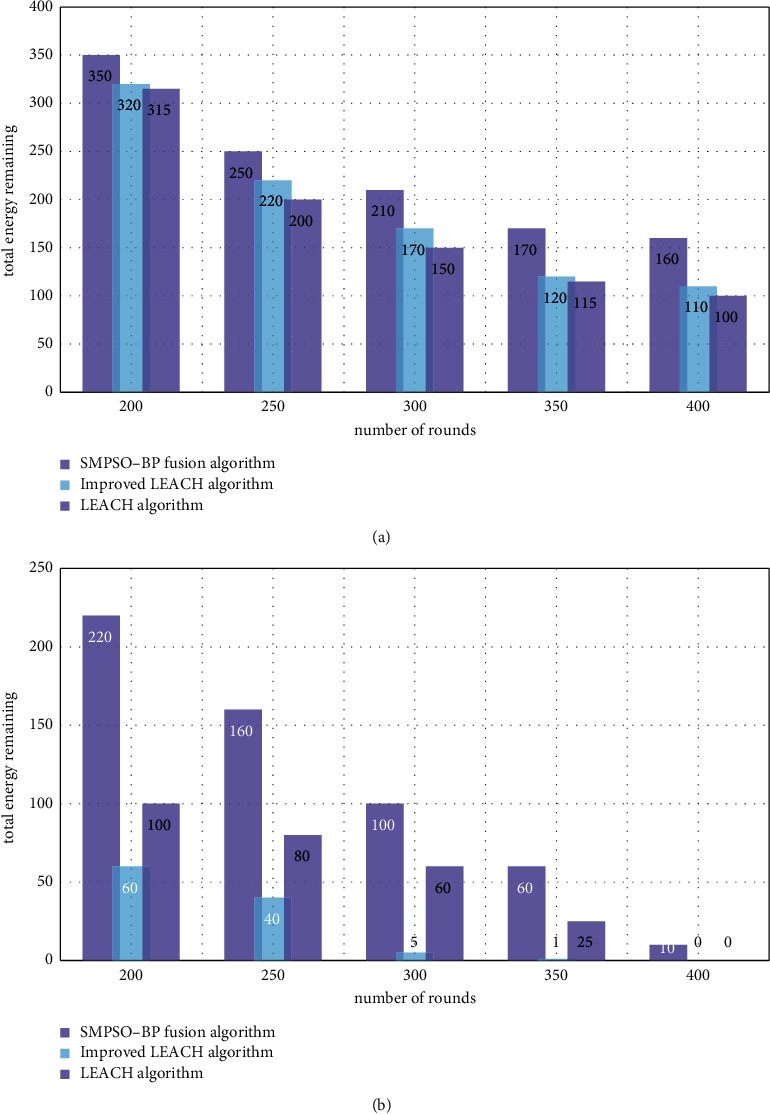
(a) The remaining energy of the whole network of the base station in the node area. (b) The remaining energy of the entire network of the base station outside the node area.

Figure 7 shows the remaining energy of the whole network when the base station is in the node area, and the remaining energy of the whole network for the three protocols in the initial state is the same. The vertical axis in Figure 7 represents the total energy remaining in the network, and the horizontal axis represents the number of rounds performed. The steep energy curves of the LEACH algorithm and the improved LEACH algorithm indicate that the energy consumption per round is more, while the corresponding SMPSO-BP algorithm protocol energy curve is the flattest, indicating that the energy consumption per round is smaller than the other two. As the round increases, the nodes far from the base station in LEACH begin to die, resulting in a reduction in the number of nodes and a reduction in energy consumption. In this experiment, the SMPSO-BP algorithm, the LEACH algorithm, and the improved LEACH algorithm are simulated and compared, and the overall energy consumption of the network is analyzed. The results are shown in Figure 7.

The node death time in the improved LEACH algorithm is later than that in the LEACH algorithm. The SMPSO-BP algorithm does not need to select a new cluster head every round. The clustering is uniform, the position and residual energy of the candidate nodes are considered when the cluster head is rotated within the cluster, and the cluster head communicates with the base station in multiple hops. These measures make the SMPSO-BP algorithm consume less energy in a single round. The residual energy of the whole network is higher than that of the LEACH algorithm and the improved LEACH algorithm. Regarding the remaining energy of the entire network when the base station is outside the node area, due to the increase in the communication distance, the energy consumed by the three protocols in each round increases. The improved LEACH algorithm has the greatest impact because all nodes need to send messages to the base station. In the SMPSO-BP algorithm, the cluster head uses multihop to transmit data to the base station, reducing the loss caused by the long-distance communication.

## 5. Conclusion

The emergence of neural network data fusion technology can better and more comprehensively simulate the cognitive ability of the sensor node ad hoc network. Its application will greatly improve the intelligence level of sensor network applications, reduce node energy consumption, and effectively prolong the lifetime of sensor networks. Three kinds of routing nodes are analyzed by the convergence speed of node data and the survival rate of nodes when data transmission is increased, and the SMPSO-BP algorithm supported by neural network is compared with other algorithms. The convergence of the SMPSO-BP algorithm and the number of surviving nodes during the operation of the wireless sensor network are all due to the other two algorithms, and it can avoid falling into the local optimum. And the cluster head election process is more reasonable, which can achieve energy balance to a certain extent. The data fusion mechanism of the SMPSO-BP algorithm does reduce the amount of data transmission during the operation of the wireless sensor network, reduce the energy consumption of nodes, and achieve the purpose of extending the network life cycle. It is believed that the energy consumption of wireless sensor network routing nodes can be better reduced based on the neural network data fusion mechanism algorithm. Wireless communication is realized through the automatic data exchange of sensors, which improves the efficiency of data transmission, provides convenience for people's daily life, and has a positive impact.

## Figures and Tables

**Table 1:** Routing classification comparison.

Algorithm	SPIN	GAF	LEACH
Topology	Flat	Location	Level
Energy saving	Have	Have	Generally
Network life	Long	Long	Long
Data fusion	Have	None	Have
Extensibility	Generally	It is good	It is good

**Table 2:** Experimental parameter value settings.

Parameter	Parameter value
The number of sensor nodes	1000
Node initial energy	0.8 J
Perceived data interval	0.5 s
Data transmission energy consumption	50 nJ/bit
Receive data energy consumption	35 nJ/bit

**Table 3:** Comparison of operation time of different algorithms.

Algorithm	Operation time/s
LEACH algorithm	4.13
Improved LEACH algorithm	3.89
SMPSO-BP	3.55

## Data Availability

The data used to support the findings of this study are available from the corresponding author upon request.
